# Nanostructured Gallium Nitride Membrane at Wafer Scale for Photo(Electro)catalytic Polluted Water Remediation

**DOI:** 10.1002/advs.202205612

**Published:** 2022-12-18

**Authors:** Huafan Zhang, Jung‐Hong Min, Tae‐Hoon Chung, Kwangjae Lee, Paulraj Gnanasekar, Jung‐Wook Min, Tae‐Yong Park, Yue Wang, Tien Khee Ng, Udo Schwingenschlögl, Qiaoqiang Gan, Boon S. Ooi

**Affiliations:** ^1^ Photonics Laboratory, Computer, Electrical, and Mathematical Sciences and Engineering Division (CEMSE) King Abdullah University of Science and Technology (KAUST) Thuwal 23955‐6900 Saudi Arabia; ^2^ Currently with the Nanophotonic Device Research Center Korea Photonics Technology Institute (KOPTI) Gwangju 61007 Republic of Korea; ^3^ Light Source Research Division Korea Photonics Technology Institute (KOPTI) Gwangju 61007 Republic of Korea; ^4^ Physical Science and Engineering Division (PSE) King Abdullah University of Science and Technology (KAUST) Thuwal 23955‐6900 Saudi Arabia; ^5^ Currently with the Department of Electrical Engineering Stanford University Stanford CA 94305 USA; ^6^ Physical Science and Engineering Division (PSE) King Abdullah University of Science and Technology (KAUST) Thuwal 23955‐6900 Saudi Arabia

**Keywords:** III‐nitride alloy, membrane, nanoporous, photocatalysis, photoelectrochemistry

## Abstract

Photo(electro)catalysis methods have drawn significant attention for efficient, energy‐saving, and environmental‐friendly organic contaminant degradation in wastewater. However, conventional oxide‐based powder photocatalysts are limited to UV‐light absorption and are unfavorable in the subsequent postseparation process. In this paper, a large‐area crystalline‐semiconductor nitride membrane with a distinct nanoporous surface is fabricated, which can be scaled up to a full wafer and easily retrieved after photodegradation. The unique nanoporous surface enhances broadband light absorption, provides abundant reactive sites, and promotes the dye‐molecule reaction with adsorbed hydroxyl radicals on the surface. The superior electric contact between the nickel bottom layer and nitride membrane facilitates swift charge carrier transportation. In laboratory tests, the nanostructure membrane can degrade 93% of the dye in 6 h under illumination with a small applied bias (0.5 V vs Ag/AgCl). Furthermore, a 2 inch diameter wafer‐scale membrane is deployed in a rooftop test under natural sunlight. The membrane operates stably for seven cycles (over 50 h) with an outstanding dye degradation efficiency (>92%) and satisfied average total organic carbon removal rate (≈50%) in each cycle. This demonstration thus opens the pathway toward the production of nanostructured semiconductor layers for large‐scale and practical wastewater treatment using natural sunlight.

## Graphical abstract

A nitride semiconductor nanostructured membrane is fabricated for a solar‐driven dye degradation utility. The reusable wafer‐scale membrane operates stably at outdoor with an outstanding dye degradation efficiency of >92%, providing satisfied and repeatable total organic carbon removal rate of ≈50%. The pathway for practical wastewater treatment using natural sunlight is thus demonstrated.

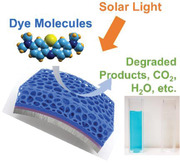

## 1 Introduction

Organic dyes are widely utilized in textile, cosmetic, and leather industries, and significant amounts are discharged in industrial wastewater, which results in considerable water contamination. Because of their toxicity and nonbiodegradability, the aquatic ecosystem can be adversely affected.^[^ ^1^ ^]^ Therefore, removing organic dyes from wastewater is imperative for a sustainable future. Various conventional methods have been used to treat these pollutants, including physicochemical techniques, such as sedimentation, physical adsorption, and filtration, and other biological techniques, such as microbial degradation.^[^ ^2^ ^]^ However, they are often inefficient since the dye molecules can only be separated or phase‐converted without being split into unharmful small molecules through physicochemical approaches, while biological degradation suffers from long reaction times ranging from weeks to even months.^[^ ^1^ ^]^ Over the years, the catalytic (electrocatalytic and photocatalytic) process of degrading dye molecules has drawn significant attention. On the downside, the well‐known process has drawbacks such as high power consumption and low degradation rates, limiting the performance of the electrocatalytic dye. Also, photocatalysis often relies on nanoparticles of powder‐based catalysts, which require post‐treatment to separate the catalysts from the remediated water and unevitably face recycle loss in the process.^[^ ^3^ ^]^ Therefore, immoblizing effective catalysts can be an easy way to resolve the postseparation issue.^[^ ^3^, ^4^ ^]^ However, simply loading the powder‐based catalysts on solid substrates still cause concerns on the stability of the fabricated photoelectrodes. It requires binder materials to further enhance the immobilization.^[^ ^3b^ ^]^

On the contrary, well‐crystallized semiconductor‐based photoelectrodes may overcome the above‐mentioned issues. Photoelectrocatalysts demand less electrical power input, is relatively efficient, chemically inert, and can be easily removed from wastewater following the reaction.^[^ ^5^ ^]^ Semiconductor catalyst materials include metal oxides, such as titanium dioxide (TiO_2_),^[^ ^6^ ^]^ tungsten oxides (WO_3_),^[^ ^7^ ^]^ and so on.^[^ ^5^, ^8^ ^]^ However, most of these metal oxides only absorb UV light, which takes up only a small amount of the solar spectrum. Hence, many oxides‐based catalysis prototypes require additional UV illumination sources for operation.^[^ ^9^ ^]^ Group‐III‐nitrides are prospective candidates for dye degradation among the wide range of semiconductors. Given the merit of their tunable bandgaps and attractive band edge positions, the materials can absorb a broad range of solar spectrum and drive the degradation reaction of the dye pollutants.^[^ ^10^ ^]^ In addition, the relatively mature growth technique and the possibility of a wafer‐scale production make them promising for practical wastewater pollutant treatments.

In this work, gallium nitride is proposed as a representative semiconductor material for nanostructured membrane photoelectrodes. The GaN membranes were exfoliated by a three‐step method: 1) porosification, 2) layer regrowth, and 3) membrane exfoliation using a stressor layer. The obtained GaN membranes have unique nanoporous (NP) top surfaces, of which the thickness can be tuned during the electrochemical porosification process. These novel nanostructured GaN membranes were applied for photocatalytic/photoelectrocatalytic dye degradation, using methylene blue (MB) as a representative dye pollutant broadly present in wastewater. Laboratory tests and rooftop field tests were conducted using small GaN membranes (0.5 cm^2^), and wafer‐scale GaN membranes (2 inch), respectively. The exfoliated NP membranes exhibit good dye degradation efficiency and stability because of their enlarged surface reactive area, improved charge carrier transportation, and promoted light absorption in the visible spectrum.

## 2 Results and Discussion

### 2.1 GaN Membrane Preparation

**Figure 1:**
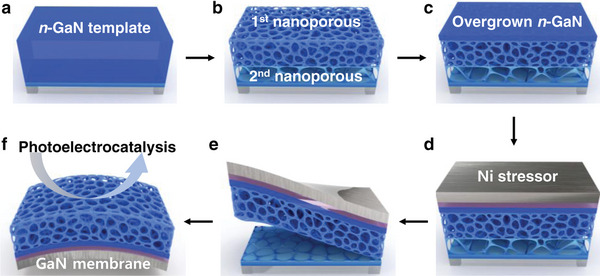
Schematic illustration of the GaN membrane with nanoporous (NP) surfaces for the photoelectrocatalysis. a) Initial thin film (TF) n‐GaN template. b) NP GaN after the two‐step porosification. c) Planarization of the NP GaN by MBE‐overgrowing n‐GaN. d) Formation of Ni stressor. e) Exfoliation. f) Exfoliated GaN membrane with a nanostructure.

The representative manufacturing process of GaN membranes with NP structures is shown in **Figure** **1**. We first formed NP structures with different porosity using a two‐step electrochemical (EC) etching of n‐GaN thin film (TF) templates (Figure 1b). The obtained NP structures were planarized by overgrowing n‐GaN through molecule beam epitaxy (MBE) (Figure 1c). After that, a tensile‐stressed Ni layer (Ni‐stressor) was applied to lift off the membrane (Figure 1d) by matching the energy release rate of the Ni stressor to the interfacial fracture toughness of the 2nd NP layer. Therefore, the exfoliated membrane consists of the MBE‐overgrown GaN with 1st NP layer (Figure 1e). Photo(electro)catalytic wastewater remediation was demonstrated by GaN membranes with various thicknesses of NP structures, as shown in Figure 1f.

#### 2.1.1 Electrochemical Porosification

**Figure 2:**
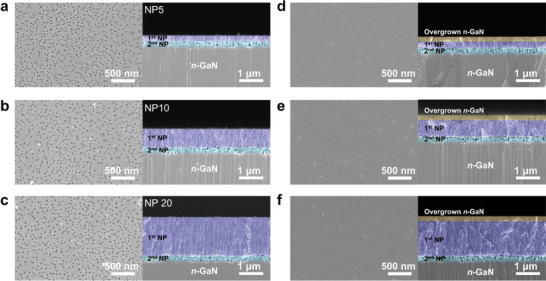
Controllable NP and its planarization by overgrowing n‐GaN layers. Representative SEM images of a–c) the NP GaN with different thicknesses and d–f) the corresponding planarized n‐GaN layers with the two‐step NP structures.

The NP GaN samples after the two‐step EC etching were shown in **Figure** **2** and Figure S1 (Supporting Information). In order to engineer the NP structures, we tuned the applied voltage and time for the EC etching in oxalic acid. Details are provided in the Materials and methods section.^[^ ^11^ ^]^ The thickness of the 1st NP layer was controlled by the EC etching duration, which was ≈250, 800, and 1600 nm for 5 min, 10 min, and 20 min etching, respectively. The surface pore size was not obviously affected by the etching time, as shown in Figure 2a–c. The 2nd NP with high porosity of the same thickness of ≈250 nm was formed through a high etching bias at constant etching time (5 min). The definition of porosity here is the ratio of voids over the total area. The 2nd Np layer can be a weakened interface for later exfoliation.

Since the thicknesses of the NP layers are determined by the etching time of the 1st NP, the obtained final NP structures were labeled accordingly, respectively, namely NP5 (5 min etching), NP10 (10 min etching), and NP20 (20 min etching).

#### 2.1.2 MBE Overgrowth for Planarization

Leveraging on the sufficient surface area of the 1st NP, n‐GaN layers were conveniently overgrown through MBE to planarize the 1st NP structures (Figure S1, Supporting Information, Figure 2d–f). The overgrown layers are well‐continuous and planarized with a thickness of ≈250 nm. The MBE growth conditions are described in the Experimental Method section.

#### 2.1.3 Exfoliation of GaN Layers with Nanoporous Structures

**Figure 3:**
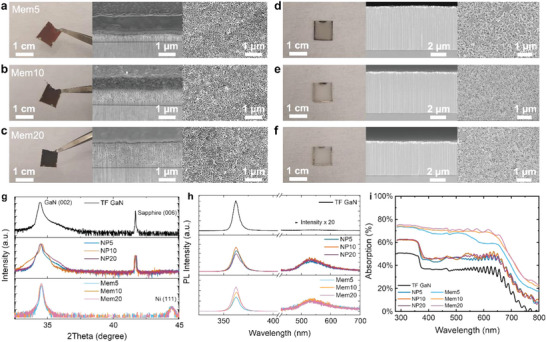
GaN membranes with NP through Ni stressor‐based exfoliation. a–c) Representative digital camera images and corresponding FIB‐SEM images of GaN membranes with a variety of thicknesses of the NP. d–f) The corresponding substrate sides after exfoliation. g) Results of XRD *ω*–2*θ* scan. h) Results of PL measurements and i) UV–vis absorption spectra.

We applied Ni stressors to peel off the planarized n‐GaN layer with the 1st NP.^[^ ^12^ ^]^ According to the Griffith's criterion, a fracture can be initiated and propagated when sufficient external energy is applied to the internal crack. The critical energy release rate determines the energy for the starting point of the fracture propagation. In our case, we intentionally formed a large volume of the voids through the 2nd NP corresponding to the internal crack. The 2nd NP acts as an internal crack and significantly decreases the total interfacial fracture toughness of the GaN layer, which is initially huge (6.34 J m^−2^). In addition, the applied Ni stressors provide enough external energy in the upward direction, perpendicularly to the surface of the n‐GaN. The total energy release rate from the Ni stressor can be engineered through the residual tensile stress of Ni and its thickness. The detailed conditions are given in the Method section. By forming the Ni stressor with sufficient energy, we conveniently peeled off the GaN layers with the 1st NP through a metal tweezer. The exfoliated GaN membranes with different thicknesses of the 1st NP are shown through the representative digital camera and focus ion beam (FIB)‐assisted‐SEM images (**Figure** **3**a–c).

The exfoliated GaN membranes (Mem GaN) were named Mem5, Mem10, and Mem20 to distinguish the different thicknesses of their NP top layer. The surface area of the GaN layer of Mem5, Mem10, and Mem20 was estimated by the Brunauer–Emmett–Teller (BET) surface area measurements, which are ≈1.52, ≈6.31, and ≈8.11 m^2^ g^−1^, respectively. Details are listed in Supporting Information S2.^[^ ^13^ ^]^ The planarized regrown layer (≈200 nm) and its interface to the Ti/Ni stressor can be clearly observed. Moreover, the remaining substrate sides after exfoliation are shown in Figure 3d–f. Clearly, the remaining NP surface evidences the exfoliation interfaces at the 2nd NP layer.

### 2.2 Material Characterization

X‐ray diffraction (XRD) patterns evidence the satisfied crystallinity of the exfoliated GaN membranes, as shown in Figure 3g. The initial TF GaN has a distinct GaN (002) peak at ≈34.5°. The GaN (002) peak remains without apparent shifts for the NP GaN and Mem GaN samples. However, the broadened and asymmetric GaN (002) peaks of NP GaN and Mem GaN suggest the electrochemical porosification introduced more structural defects, in accordance with the previous PL results. Nevertheless, the narrowed GaN (002) peak of Mem GaN indicates a satisfied crystallinity of the MBE regrowth layer. The phi scans of the asymmetric GaN (102) plane of the TF GaN, NP GaN, and Mem GaN all exhibited a six‐fold symmetry, confirming the hexagonal structure of all the samples (Figure S3, Supporting Information). From the observations, it is confirmed that the electrochemical etching process formed a porous surface layer. However, it did not affect the crystalline orientation, so the consecutive grown MBE layer followed the same lattice orientation. Therefore, the exfoliated membranes, mainly the MBE regrown layer, exhibit satisfied crystallinity. Scanning trasmission electron microscopy (STEM) images and the corresponding fast Fourier transform (FFT) patterns of the Mem GaN (Mem5 as a representative) also identify the single crystallinity of the exfoliated membrane. Details are described in Supporting Information S4.

The photoluminescence (PL) spectra are shown Figure 3h. There is no noticeable change in the GaN band edge emission peak, which locates at ≈363 nm for all samples. However, the electrochemical etching process introduced more structural defects, which gave rise to a noticeable yellow luminescence peak of the PL spectra among all the NP GaN and Mem GaN samples. Those radiative surface defects may enhance visible light absorption and intra‐band carrier transition, benefiting visible‐light‐driven photocatalysis.^[^ ^10a^ ^]^ The UV–vis spectrophotometer (Figure 3i) confirmed the enhanced broadband light absorption by scattering inside the porous layer. Interestingly, the membrane can further assist in visible light absorption since the bottom Ti/Ni layer significantly reflects the photons and introduce multiple reflection and scattering within the top NP layer.

### 2.3 Membrane for Dye Degradation (Laboratory Tests)

After confirming and optimizing the fabrication process of the GaN membranes, the photoelectrocatalysis performance was evaluated and compared to the TF GaN and NP GaN samples. The principal investigation and optimization were conducted by using small‐size Mem GaN (≈0.5 cm^2^) as the working electrode in an in‐situ setup (Figure S5, Supporting Information). In this paper, MB is selected to be degraded as a commonly existing dye pollutant in wastewater. Since the MB concentration is linearly correlated to the UV–vis absorbance at ≈663.5 nm, the degradation efficiency (*η*) can be calculated by Equation 1,^[^ ^5^, ^14^ ^]^ where *C*_0_, *A*_0_ and *C_t_, A_t_* are the dye concentration (mg L^−1^) and absorbance at the beginning and after reaction time *t* (min), respectively

(1) $$\eta =\frac{C_{0}-C_{t}}{C_{0}}\times 100\%=\frac{A_{0}-A_{t}}{A_{0}}\times 100\%$$

Furthermore, the apparent rate constant (*k*_app_) can also be described by a pseudo‐first‐order kinetic model (Equation 2), i.e., the so‐called Langmuir‐Hinshelwood kinetic equation.^[^ ^5^, ^14^ ^]^ The rate constant normalized to the surface area (cm^−2.^min^−1^) can be obtained by fitting the slope of plot ln (*C_t_*/*C*_0_) versus reaction time. Fitting examples can be found in Figures S6b and S7b (Supporting Information)

(2) $$\ln\frac{C_{t}}{C_{0}}=-k_{\mathrm{app}}*A*t$$

The PEC MB degradations (5 mg L^−1^) were tested using a Xenon lamp with a calibrated illumination intensity of 100 mW cm^−2^ and an applied potential of 0.5 V versus Ag/AgCl (≈1 V vs RHE). Before each test, the sample was kept in the electrolyte under dark conditions for 30 min to ensure the surface absorption equilibrium.

This PEC condition was confirmed by a series of optimization tests described in Supporting Information S4. The dye solution presented 7% and 12% self‐degradations within 6 h under dark and illumination conditions, respectively, which are several folds lower than the degradation efficiencies when using Mem GaN as catalysts (Figure S6a,b, Supporting Information). Bias‐dependent tests were also conducted on as‐grown, NP, and Mem GaN samples (Figure S6c, Supporting Information), which identified an optimized applied potential of 0.5 V versus Ag/AgCl (≈1 V vs RHE at pH 6) for the PEC measurements. Notably, 32% of dye degradation was witnessed for the Mem20 sample in the electrocatalytic reaction. Fascinatingly, almost 65% and 91% of degradation were observed for the same testing period while using Mem20 as a photocatalyst and photoelectrocatalysts, respectively.

**Figure 4:**
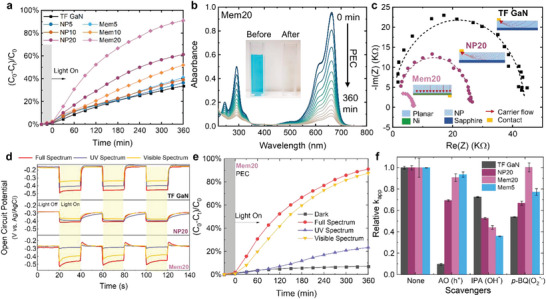
a) Photoelectrocatalytic MB degradation performance of the TF, NP GaN, and Mem GaN samples. b) Decolorizations of MB observed by a UV–vis spectrophotometer during the photoelectrocatalysis by the Mem20 sample (inset: picture of the MB solution before and after the reaction). c) Nyquist plots of TF GaN, NP20, and Mem20 samples. d) Open circuit potential of the TF GaN, NP20, and Mem20 samples under full‐spectrum, UV, and visible light illumination. e) Photoelectrocatalytic degradation performance of the Mem20 sample under different light illuminations. f) Scavenger quenching tests.

From the observations, PEC displayed excellent degradation performance and was adopted for further comparison. All the samples were examined under the settled PEC conditions for 6 h for comparison (**Figure** **4**a). The TF GaN sample can degrade 34% of the MB in the electrolyte. After various porosification, the NP GaN samples present higher degradation efficiencies that correlate to the 1st NP layer depth, and their efficiency can reach 36%, 39%, and 61% for the NP5, NP10, and NP20 photoelectrocatalyst, respectively, attributing to a larger effective surface area compared to the TF GaN sample. With an NP layer of similar thickness, the membranes show significant enhancement in degradation efficiencies compared to the NP GaN samples. Notably, the Mem20 sample can bleach more than 91% of MB from the water in 6 h and is three times superior to TF GaN. The corresponding chronoamperometry (CA), *k*_app_ fitting, and linear sweeping voltammetry (LSV) are described in detail in Supporting Information S7. Representative UV–vis absorbance spectra of the bleaching dye solution using Mem20 are demonstrated in Figure 4b, with the observable decolorization of the sampling solution before and after the reaction shown as the inset. It is clear that MB degradation was negligible for the first 30 min under the dark condition, while the concentration considerably dropped during the photoelectrocatalysis process by the Mem20 sample.

The superior PEC performance of Mem GaN over NP GaN with the same NP layer thickness may be attributed to multiple reasons. One of the reasons is the suppressed interfacial resistance and efficient carrier transportation at the back contact. Electrochemical impedance spectroscopy and open‐circuit potential measurements were conducted to investigate the carrier transportation behaviors of the GaN membrane compared to NP GaN and TF GaN.^[^ ^15^ ^]^

All the Nyquist plots (Figure 4c) showed a single semicircle curve, which indicates the absence of interfacial resistance at the NP GaN/Ni interface for efficient photogenerated carrier transportation. In addition, the smaller the arc radius for the Nyquist plots, the better the carrier transfer with a lower impedance.^[^ ^16^ ^]^ The impedance of NP20 is lower than that of the TF GaN, mainly due to the higher surface area. Since the electrolyte can immerse inside the pores and create a larger GaN/MB interface, more photogenerated carriers can access the interface in the vicinity and drive the reaction effectively.^[^ ^17^ ^]^ Meanwhile, Mem20 further suppresses the impedance by the excellent electric back contact of Ti/Ni to the entire continuous GaN layer, indicating a better potential for faster carrier transportation compared to the NP20 sample, as shown in the inset schematics.

Open‐circuit potential (OCP) measurements (Figure 4d) further confirmed the results. During the transient measurements, the open‐circuit potentials shifted to negative potentials upon turning on the illumination due to the photocarrier generation, and they shifted back when the illumination was turned off due to the discharged carriers. Different OCP decay behaviors can be witnessed for the TF GaN, NP20, and Mem20, even under the same illumination conditions. This exponential delay in TF GaN upon light off suggests rapid photogenerated carrier recombination.^[^ ^18^ ^]^ Nevertheless, the etching process may introduce more defects, leading to slower carrier recombinations in NP GaN samples but providing more adsorption and reaction sites to the dye molecules. In contrast, the OCP showed sharp decays for the membrane, indicating efficient carrier separation and fast extraction to the external circuits,^[^ ^18^ ^]^ which is most likely due to the superior electric back contact.

In addition, the OCP shifts of a sample exhibited different magnitudes in response to the full spectrum, UV spectrum, and visible spectrum, implying different photoresponses toward different wavelengths. The spectra of the three incident lights are shown in Figure S8 (Supporting Information). The OCP shifts are as expected from the PL and UV–vis absorption results (Figure 3). TF GaN and NP GaN samples have a better photoresponse to UV light over visible light, while the Mem GaN samples have a better selectivity toward the visible spectrum. Considering the structures of the membranes, the NP layer introduced higher absorption in the visible range by structural scattering and reflection, while the back metal layer further enhanced it due to negligible transmission loss and good back reflection into the NP layer. Meanwhile, the defects‐introduced radiative intra‐band transition may also contribute to a better photoresponse in the visible region. The corresponding degradation efficiencies and LSV of the membranes upon different light illumination are shown in Figure 4e and Figure S8 (Supporting Information). In accordance with the OCP results, Mem20 GaN shows a superior degradation efficiency (87%) under visible light illumination over TF and NP20 GaN in the same testing period.

Quenching tests were conducted to investigate the reaction mechanism of the membranes further. By adding various scavengers that target different reactive oxygen species (ROS), the tests identified the most effective ROS and provided other explanations for the outstanding performance of the membranes through the possible reaction pathway.^[^ ^19^ ^]^ The ROS of membranes were investigated by adding 0.01 m ammonium oxalate (AO), isopropanol (IPA), and p‐benzoquinone (p‐BQ) as scavengers for holes (*h* ^+^), hydroxyl radicals (OH*), and superoxide radicals (O_2_^*−^), respectively. The *k*_app_ was calculated by Equation 2, and the relative *k*_app_ in the presence of the scavengers is given in Figure 4f. For TF GaN, photogenerated holes play vital roles in the PEC degradation of MB, while interestingly, the OH* species are the prominent ROS for NP GaN and Mem GaN.

The EC etching process introduced more defects or vacancies on the surface of pores, and the NP structure exposed complicated tiny facets of GaN crystals in the electrolyte. These structural changes can modify the electronic structures at the surface GaN by slightly changing the surface band bending or forming bonds with ROS in the electrolyte.^[^ ^20^ ^]^ In NP GaN and Mem GaN, these defect sites may behave as active sites and bond with the OH* radicals on the surface.^[^ ^20a^ ^]^ During the reaction, the photogenerated holes can easily access the enlarged GaN/MB electrolyte interface, react with water molecules, and produce and stabilize OH* radicals at the surface of pores. On the other hand, the photogenerated electrons migrate through the external circuits to the counter electrode, react with dissolved oxygen molecules, and produce more O_2_^*−^, which can further react with water and form more OH* radicals for effective MB degradation. The generated ROS can nonselectively break the bonds of MB molecules in the electrolyte.^[^ ^19^ ^]^ From the above‐mentioned results, the possible reaction pathway explains the advanced performance of the membranes, which can be given by Equations (3), (4), (5), (6), (7), (8).

(3) $$\mathrm{GaN}+h\nu \to h^{+}+e^{-}$$

(4) $$h^{+}+H_{2}O\to {\mathrm{OH}}^{*}+H^{+}$$

(5) $$e^{-}+O_{2}\to O_{2}^{*-}$$

(6) $$O_{2}^{*-}+2H^{+}+e^{-}\to H_{2}O_{2}$$

(7) $$H_{2}O_{2}+H^{+}+e^{-}\to {\mathrm{OH}}^{*}+H_{2}O$$

(8) $$\mathrm{dye}+{\mathrm{OH}}^{*}\to \mathrm{intermediates}\to \mathrm{degradation}\;\mathrm{products}$$

It is also worth mentioning that the relative PEC degradation performance of Mem5 and Mem20 have similar responses to the three scavengers. These observations suggest that the enlarged surface area only improves the absolute PEC degradation rates, while the electronic structure modification of GaN caused by the etching‐introduced defects determines the reaction pathway.

### 2.4 Outdoor Stability Test for 2 Inch Wafer‐Scale Membrane

**Figure 5:**
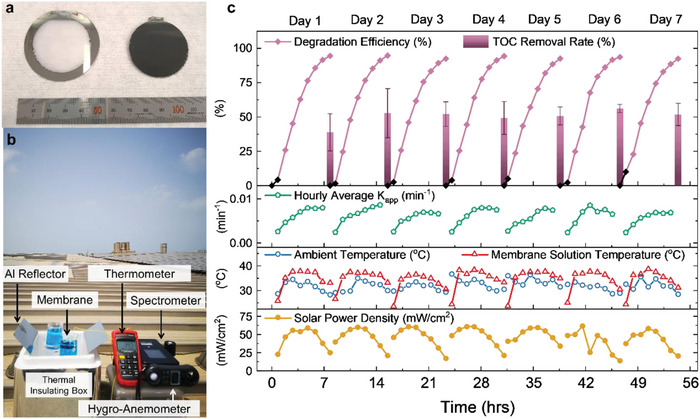
a) Picture of an exfoliated 2 inch diameter membrane and the remaining porous GaN template. b) Picture of the rooftop setup. c) Photocatalytic MB decolorization and TOC removal rates. The hourly average decolorization rates with the real‐time temperature and solar light power.

After determining and understanding the reactions using small‐size photoelectrodes, the experiment was scaled up to wafer‐level for an outdoor field test on the rooftop of an experimental building at King Abdullah University of Science and Technology, Thuwal, Saudi Arabia. The 2 inch diameter wafer‐scale membrane was obtained through the same method as the Mem20 sample, as shown in **Figure** **5**a. The manufacturing process and exfoliation are shown in Figure S9 (Supporting Information). The wafer‐scale membrane was suspended at ≈2 cm below the initial solution level in the beaker, which was placed on a holder on top of a thermal insulating box to prevent additional heating from the metallic rooftop. Two aluminum boards were attached at 45° on two sides of the box toward the sky for a more efficient reflection of solar light onto the samples. The setup (Figure 5b) was kept in the dark for at least 30 min before the daytime 7 h‐rooftop test to guarantee the surface adsorption equilibrium of the membrane. The tests were conducted over seven days under different weather conditions. The ambient temperature, solution temperature, solar light power density, relative humidity, and wind speed were monitored simultaneously with MB solution sampling at each hour.

The results are shown in Figure 5c and Supporting Information S10. The total organic carbon (TOC) removal rates were measured at the end of each test (Figure 5a). The large‐scale membrane showed excellent stability and repeatability for seven days. It degraded the MB by more than 92% and removed the TOC by an average of 50.2% in 7 h each day. The hourly average apparent degradation rate constants (calculated by Equation S10, Supporting Information) change with the solar light illumination and temperature over a day. The membrane can operate even under relatively weak light intensity of ≈20 mW cm^−2^, which is also proved by an indoor light intensity‐dependent photocatalysis experiment, as shown in Supporting Information S8. During the rooftop measurement, the average degradation rates increased to a peak of ≈7.5 × 10^−3^ min^−1^ at around 13:00 to 14:00, when the solar illumination and temperature reached their maximum in a day. The onsite rooftop measurement proves the potential of scaling up the membrane photocatalysts for a practical dye‐polluted water treatment system using natural solar energy.

## 3 Conclusion

In summary, a scalable single‐crystalline GaN membrane with a controllable nanoporous surface features was fabricated and demonstrated for photo(electro)catalytic organic dye degradation in natural sunlight. The increased surface reactive area provided enriched reaction sites and enhanced visible light absorption, significantly enhancing the dye degradation efficiency. The reaction pathway was investigated in depth by quenching tests. The reaction mechanism is related to the enriched active sites, such as surface defects during the formation of NP structures. The encouraging performance of the small‐sized membrane can be further scaled up to a wafer scale. The seven‐cycle long‐term rooftop photocatalysis experiments prove the excellent stability and repeatability of the wafer‐scale membrane, which can decolorize ≈92% of MB and achieve an average TOC removal rate of ≈50% under natural solar illumination. The unique semiconductor membrane exhibited promising stability and efficiency for wastewater remediation applications without the assistance of any co‐catalysts or additional additive chemicals in the dye solutions. Further performance enhancement can be realized by customizing the nitride membrane photoelectrodes through the doping levels and compositions of the MBE overgrown layer or by optimizing the dye solutions, such as tuning the pH, conductivity and adding oxidants such as hydrogen peroxide.^[^ ^5^, ^21^ ^]^ Other organic contaminant removal reactions may also be explored. The proof‐of‐concept laboratory tests and rooftop prototype deployment suggest the excellent scalability and stability of the nanostructured semiconductor membranes for practical solar light‐driven and visible‐light‐driven organic pollutant remediation from wastewater, which expands the clean energy conversion and environmental sustainability applications of conventional semiconductors with nanostructures.

## 4 Experimental Section

### Electrochemical Porosification

The initial metal‐organic chemical vapor deposition (MOCVD)‐grown thin film (TF) n‐GaN consists of a 5 µm‐thick n‐GaN layer (≈6 × 10^18^ cm^−3^ doping concentration) on a 2 µm‐thick undoped‐GaN buffer layer on sapphire substrates. The two‐step NP structures were prepared through electrochemical (EC) etching of the TF n‐GaN. An electrode was formed by indium soldering at the edge of the template. Afterward, the TF n‐GaN and a Pt counter electrode were connected to the positive and negative electrodes in 0.3 M oxalic acid solution.^[^ ^11^, ^16^ ^]^ To form two‐step NP structures, 11 and 21 V input voltages were applied with 50 mA for compliance for the 1st NP and 2nd NP layer, respectively.

### MBE Overgrowth for Planarization

The overgrowth of n‐GaN on the NP GaN was performed through molecular beam epitaxy (MBE) (VEECO GEN 930). Ga flux and N_2_ plasma (200 W with 0.6 sccm for N_2_ flow) were used for the Ga and N sources. A Si cell was heated up to 1180 °C for n‐type dopants. The NP GaN templates were loaded in a load lock chamber and baked out at 200 °C for 1 h. Further outgassing was performed in a buffer chamber at 600 °C for 3 h. Afterward, the samples were loaded in the main chamber and heated up to 200 and 700 °C in order with 5 and 20 °C min^−1^ ramping rates, respectively. Finally, a 200 nm thick n‐GaN layer was directly overgrown on the two‐step NP without a low‐temperature buffer layer by opening all related source cells simultaneously.

### Ni Stressor‐Based Exfoliation

An electroplating process was applied to form the Ni stressor.^[^ ^12^ ^]^ Before electroplating the Ni layer, Ti/Ni layers (50 nm thick for small‐size samples and 100 nm thick for wafer‐scale samples) were directly deposited onto the overgrown n‐GaN layer as an adhesion layer and seeding layer. Subsequently, the samples and a Ni plate were dipped into a 55 °C NiSO_4_‐based solution and connected to a negative and a positive electrode, respectively. The electroplating process was performed at ≈2.4 V for small‐size samples and at ≈3.4 V for the wafer‐scale samples. After forming the Ni stressors, the GaN layer with the 1st NP was easily exfoliated by gently pulling the Ni stressors with a tweezer.

### Materials Characterization

Scanning electron microscopy (SEM) images were acquired by Zeiss Merlin scanning electron microscope. X‐ray diffraction (XRD) patterns were acquired by a Bruker D8 Ultra diffractometer equipped with a monochromator using Cu K*α* (*λ* = 1.5405 Å) radiation. Photoluminescence (PL) spectroscopy was examined using the WiTec Apyron PL system under an excitation wavelength of 325 nm. For UV–vis absorption measurements of solid samples, the absorption (*A*) is obtained by *A = 1 – R – T*, where *R* is the reflectance and *T* is the transmittance. The diffusive reflectance is measured by using an Al mirror (Thorlabs, PF10‐03‐F01) as an absolute reflectance reference. The transmittance is measured by using ambient air as a reference. Both reflectance and transmittance measurements were conducted by using a built‐in integrating sphere in Shimadzu UV‐3600.

### Photoelectrochemical Characterization

The laboratory tests of photoelectrochemical characterizations were conducted in a three‐electrode cell in 5 mg L^−1^ methylene blue solution, containing the sample working electrode, a Pt coil counter electrode, and an Ag/AgCl reference electrode. The analysis was carried out with a reversible hydrogen electrode (RHE) correction using the Nernst equation *E*_RHE_ = *E*_0_ + *E*_Ag/AgCl_ (0.198 *V*) + 0.059 × pH. The measurements were carried out by a Biologic SP‐150 Potentiostat. A 100 W Xe lamp (Asahi Spectra) was used as the light source. The light intensity at the sample surface was calibrated to be 100 mW cm^−2^ by a silicon photodiode (Thorlabs PM100D with S120VC). A 2 inch Al mirror (Thorlabs, PF20‐03‐F01), short pass dichroic mirror (Thorlabs, DMSP425L), or long pass dichroic mirror (Thorlabs, DMLP425L) was installed to change the full spectrum illumination to the visible spectrum or UV spectrum, respectively. Before each dye degradation reaction, the samples were kept in the electrolyte in the dark for 30 min to ensure surface adsorption equilibrium. The detailed laboratory setup is shown and described in Figure S1 (Supporting Information). For dye degradation measurements, 2 mL of the sampling dye solution flowed into a UV cuvette in the UV–vis spectrophotometer (Shimadzu UV‐3600), and the corresponding absorbance was measured in the transmittance mode using a built‐in integrating sphere. The MB concentration of the sampling solution is linearly correlated to the absorbance; thus, the relative degradation efficiency can be calculated.^[^ ^22^ ^]^ Details are listed in the Supporting Information.

### Rooftop Field Test

The outdoor experiments were conducted using a 2 inch GaN membrane for seven days during the daytime, from 9:00 to 16:00, in April 2022, on the rooftop of Ibn Sina building at King Abdullah University of Science and Technology, Thuwal, Saudi Arabia. The total organic carbon (TOC) of the dye solution was tested before and after the photocatalytic process by a TOC analyzer (TOC‐L, Shimadzu) equipped with an autosampler (ASI‐L, Shimadzu) in the nonpurgeable organic carbon (NPOC) mode. The TOC calibration was conducted by creating a gradient sequence (0–2 ppm) using a TOC standard solution (Sigma‐Aldrich, 1000 ppm).

## Conflict of Interest

The authors declare no conflict of interest.

## Supporting information

Supporting Information

## Data Availability

The data that support the findings of this study are available from the corresponding author upon reasonable request.
