# Supplementary material for: Nanostructured Gallium Nitride Membrane at Wafer Scale for Photo(Electro)catalytic Polluted Water Remediation
Source: Adv Sci (Weinh). 2022 Dec 18;10(6):2205612. doi: 10.1002/advs.202205612 (PMC9951313; doi:10.1002/advs.202205612)
Supplement: Supplementary file 1 — Supporting Information [file ADVS-10-2205612-s001.pdf]

Supporting Information

Title Nanostructured gallium nitride membrane at wafer scale for photo(electro)catalytic polluted water remediation

Huafan Zhang, Jung-Hong Min, Tae-Hoon Chung, Kwangjae Lee, Paulraj Gnanasekar, Jung-Wook Min, Tae-Yong Park, Yue Wang, Tien Khee Ng, Udo Schwingenschlögl, Qiaoqiang Gan, Boon S. Ooi *

† These authors contributed equally to this work.

H. Zhang, J-H. Min, P. Gnanasekar, J-W. Min, T-Y. Park, Y. Wang, T. K. Ng, and B. S. Ooi
Photonics Laboratory, Computer, Electrical, and Mathematical Sciences and Engineering Division (CEMSE), King Abdullah University of Science and Technology (KAUST), Thuwal 23955-6900, Saudi Arabia
E-mail: boon.ooi@kaust.edu.sa

J-H. Min
Currently with the Nanophotonic Device Research Center, Korea Photonics Technology Institute (KOPTI), Gwangju 61007, Republic of Korea

T-H. Chung
Light Source Research Division, Korea Photonics Technology Institute (KOPTI), Gwangju 61007, Republic of Korea

K. Lee
Physical Science and Engineering Division (PSE), King Abdullah University of Science and Technology (KAUST), Thuwal 23955-6900, Saudi Arabia,
Currently with the Department of Electrical Engineering, Stanford University, Stanford, CA 94305, United States of America

U. Schwingenschlögl, and Q. Gan
Physical Science and Engineering Division (PSE), King Abdullah University of Science and Technology (KAUST), Thuwal 23955-6900, Saudi Arabia

S1. 2-step nanoporous and its planarized samples

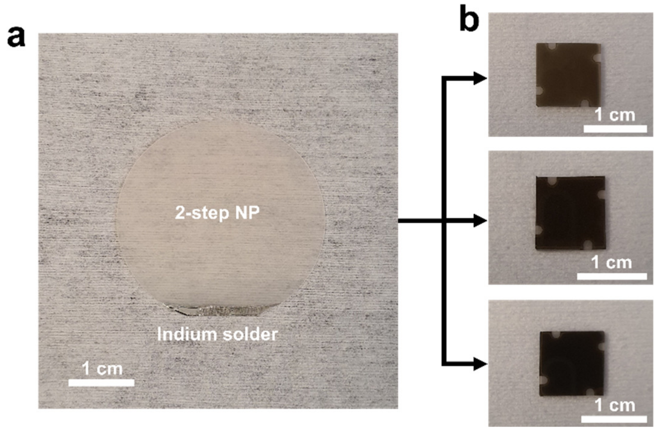

Figure S1. (a) A representative digital camera image of the 2-step nanoporous (NP) GaN. (b) Digital camera images for the planarized n-GaN layers of NP5, NP10, and NP20 (top to bottom, respectively.

S2. BET Measurement results.

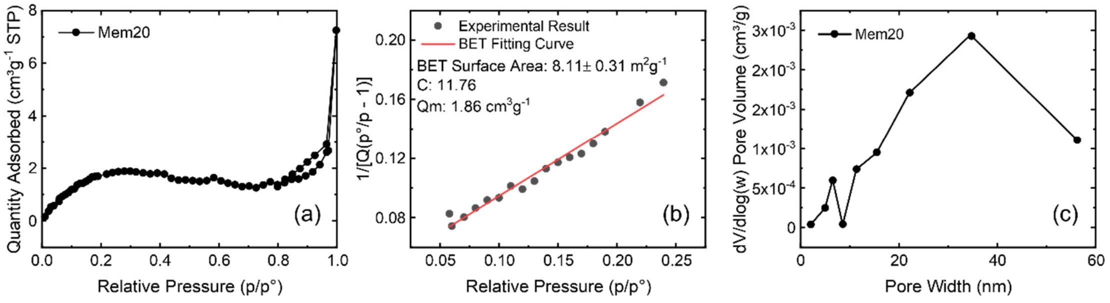

Figure. S2. BET surface area analysis of Mem20. (a) Nitrogen adsorption-desorption isotherm. (b) BET surface area analysis. (c) BJH pore size distribution analysis.

The nitrogen adsorption-desorption isotherms (**Figure. S2a**) was measured by Micrometrics ASAP2420 instrument at the liquid nitrogen temperature (77K). Prior to the measurements, the samples were degassed in a vacuum at 90 ºC for 1 hour, then ramped up to 300 ºC for 3 hours, and finally outgassed to 1 mTorr. The BET surface area (**Figure. S2b**) and pore size distribution curves (**Figure. S2c**) (based on Barret, Joyner, and Helenda (BJH) model with Faas correction) were analyzed to determine the specific surface area and pore volume of the GaN layer. The results are shown in **Figure. S2** using Mem20 as a representative since the sample has the clearest isotherm behavior, BET fitting, and typical pore size distribution. The isotherm exhibits a mixed type II and type III behavior, indicating relatively weak absorbate-absorbent interactions and varied pore size distributions. It matches with the observation from the SEM cross-section images (**Figure. 3a-3c**), the surface NP layers of the membrane contain complicated pore shapes, with different sizes of cylindrical branches and partially blocked pores with a size ranging from a few nanometers to up to a few tens of nanometers.[1]

The estimated BET surface area values of all the Mem GaN samples are listed in Table S2.

Table. S2. The estimated BET Surface Area of Mem GaN.

Sample	BET Surface Area m^2g^{-1}	Error m^2g^{-1}
Mem5	1.52	0.38
Mem10	6.31	0.39
Mem20	8.11	0.31

S3. XRD in-plane φ scan of GaN(102) plane

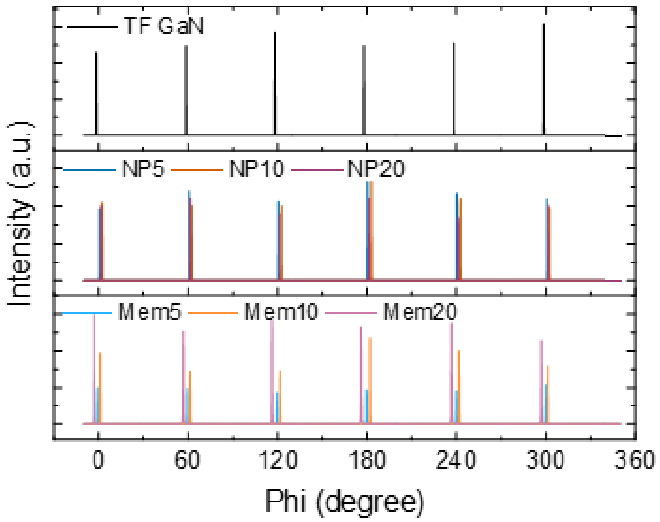

Figure S3. XRD in-plane φ scan of GaN(102) plane.

S4. STEM Images of Mem GaN

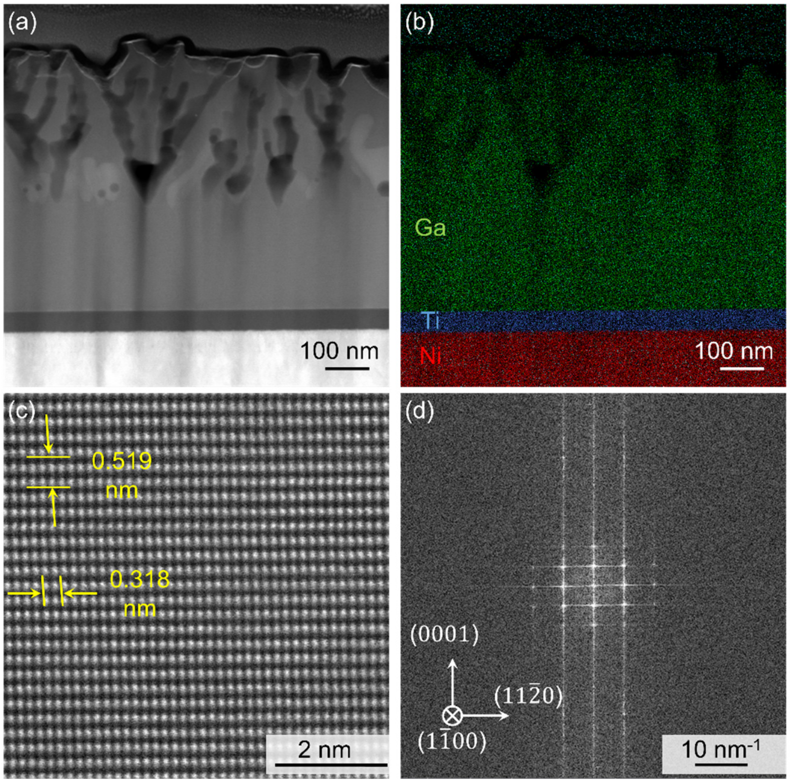

Figure. S4. (a) Scanning transmission electron microscopy (STEM) images of a Mem GaN sample, using Mem 5 as the representative. (b) The corresponding EDX elemental mapping of (a). (c) The corresponding high-resolution (HR)-TEM image. (d) The corresponding FFT pattern of (c).

TEM measurement on the Mem5 sample. The sample was prepared as TEM lamellar by a dual beam focus ion beam (FIB)/ SEM (FEI Helios). The Scanning transmission electron microscopy (STEM) images of the Mem 5 lamellar are acquired by FEI Titan Themis STEM. Figure. S4 (a) shows the TEM images of the membrane. The structure clearly consists of the NP GaN layer, the continuous MBE overgrown layer, the Ti adhesion layer, and the Ni stressor. The corresponding EDX elemental mapping were shown in Figure. S4(b). From the HR-TEM images (Figure. S4(c)), the continuous MBE epitaxy layer on the NP layer follows the same orientation as the initial NP template and exhibits well-crystallized atomic arrangements. The measured lattice constant of the GaN layer is ~ 0.519 nm along the c-axis and ~ 0.318 nm along the a-axis, matching the lattice constant in the literature.[2] The fast Fourier transforms patterns are shown in Figure. S4 (d), revealing the coherency of the wurtzite crystal structure of the MBE overgrown layer and the NP layer.[3]

S5. Laboratory PEC setup

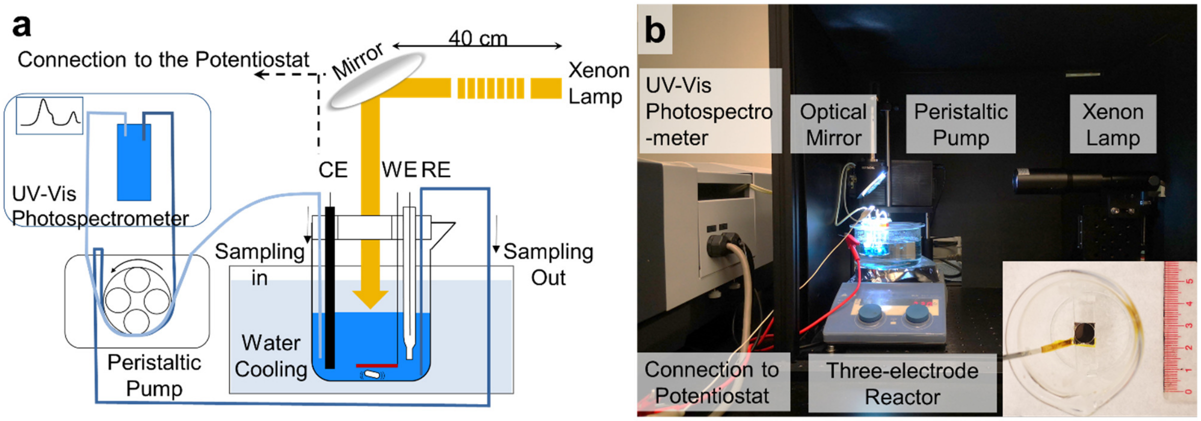

Figure S5. Photoelectrocatalysis setup with *in-situ* UV-Vis spectrophotometry. (a) Schematics. (b) Picture of the actual setup.

The samples were connected to an Al belt using Indium contact as a working device for all the electrochemical measurements, as shown in the inset. The electric contact was sealed with Kapton tape and then fully covered with PDMS to ensure only the reactive surface is exposed to the illumination and contact with the electrolyte. During the experiment, the sample was fixed by a PMMA framework to ensure the sample surface immersing in the electrolyte at a distance of 1 cm. (PDMS and PMMA were tested prior to the measurements to ensure there is no reaction with Methylene blue.) The three-electrode PEC reactor consists of a 0.5 cm^2 sample, Ag/AgCl (Sat. KCl), and a Pt coil as the working electrode, reference electrode, and counter electrode, respectively. The working electrode was immersed in the electrolyte at a ~ 1 cm distance to the electrolyte surface. The electrochemical measurements were conducted by a potentiostat (EC-lab SP150). A two-inch Al mirror (Thorlabs PF20-03-F01) was installed at 45° with a distance of 14 cm above the sample surface to reflect the incoming light from the Xenon lamp (Hal-320), which locates 30 cm away from the reactor. The light intensity at the sample surface was calibrated to be 100 mW/cm^2 by a silicon photodiode (Thorlabs PM100D with S120VC). This optical path was designed to prevent undesired heating of the electrolyte. Also, the Al mirror can be easily replaced by a 2-inch short-pass dichroic mirror (DMSP425L) or long-pass dichroic mirror (DMLP425L) to change the full spectrum illumination to visible spectrum or to UV spectrum, respectively. Before each dye degradation reaction, the samples were kept in the electrolyte in the dark for 30 min to ensure surface adsorption equilibrium. The electrolyte was mixed vigorously during the reaction by a magnetic stirrer and kept circulating from the cell to a sampling vial (2 mL) in a UV-Vis spectrophotometer (Shimadzu UV-3600)

at a speed of 10 mL/min. This in-situ UV-Vis spectrophotometry measured the spectra of the sampling dye solution at an interval of every 30 min.

S6. PEC condition optimization

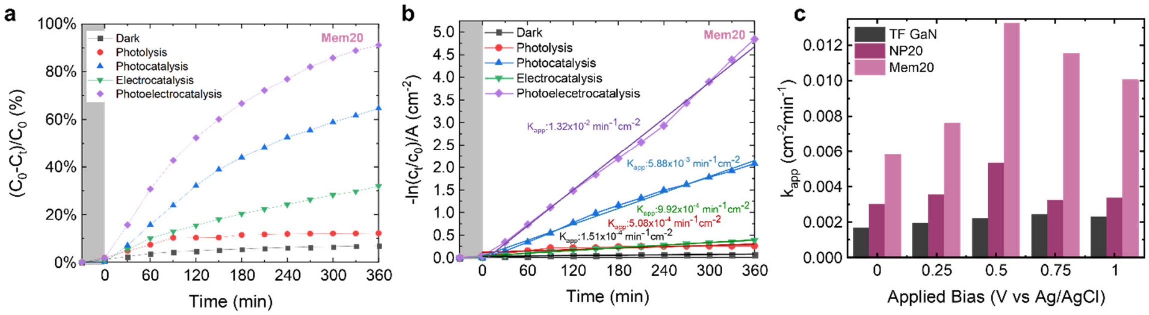

Figure S6. PEC condition optimizations. (a) MB self-degradation in dark, under light (photolysis), photocatalysis (PC), electrocatalysis (EC), and photoelectrocatalysis (PEC) of membrane 20-min samples. (b) Fitting curves of the calculated apparent rate constants. (c) Bias-dependent tests of planar GaN, NP20, and Mem20 samples.

Bias-dependent tests were conducted on planar, porous, and membrane samples, which identified an optimized applied potential of 0.5 V vs. Ag/AgCl (~ 1 V vs. RHE at pH 6) for the PEC measurements. The pH of the solution was almost constant at 6 without obvious changes throughout the whole reaction. The degradation rates were slightly lower when applying an applied potential higher than 0.75 V vs. Ag/AgCl (~ 1.3 V vs. RHE), which may be attributed to the competative reaction of oxygen evolution reaction (OER) (standard potential at ~ 1.23 V vs. RHE).[4] The photogenerated carriers swiftly react with the water molecules and the produced reactive oxygen species (ROS) can also combine with the excess carriers. Thus, OER exceeds MB degradation reaction in the electrolyte.

S7. Supplementary PEC data.

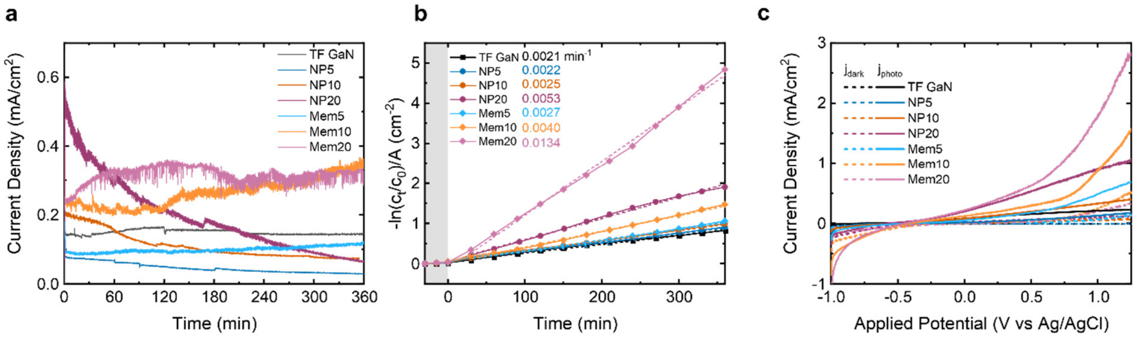

Figure S7. (a) Chronoamperometry (CA) during the PEC tests, (b) the apparent degradation rate constant fitting, (c) linear sweeping voltammetry (LSV) of different samples.

The CA of planar GaN was stable during the 6-hour test. The CAs for all the NP GaN samples showed different extents of degradation, which may be caused by surface oxidation of the porous samples. However, the CA for membrane samples is prone to an increasing trend which may partially be due to the increased incident light intensity penetrated through the quickly bleached electrolyte and reached the sample surface, or maybe because the fast carrier extraction through the Ni back contact suppresses the undesired surface oxidations. On the other hand, specifically, an upward trend can be observed at the beginning stage of CA for Mem20, which may be attributed to the slow activation of the surface reactive sites, since the sample has the thickest surface NP layer.

From the linear sweeping voltammetry, it is clear that the NP and Mem samples have a better photoresponse than the planar GaN despite the increased dark currents. Considering the PEC degradation results, although planar GaN has a larger photocurrent compared to porous 5 min and membrane 5 min samples, the actual degradation rates are limited, which suggests a low conversion efficiency of photogenerated carriers to reactive oxide species.

S8. Different light illuminations

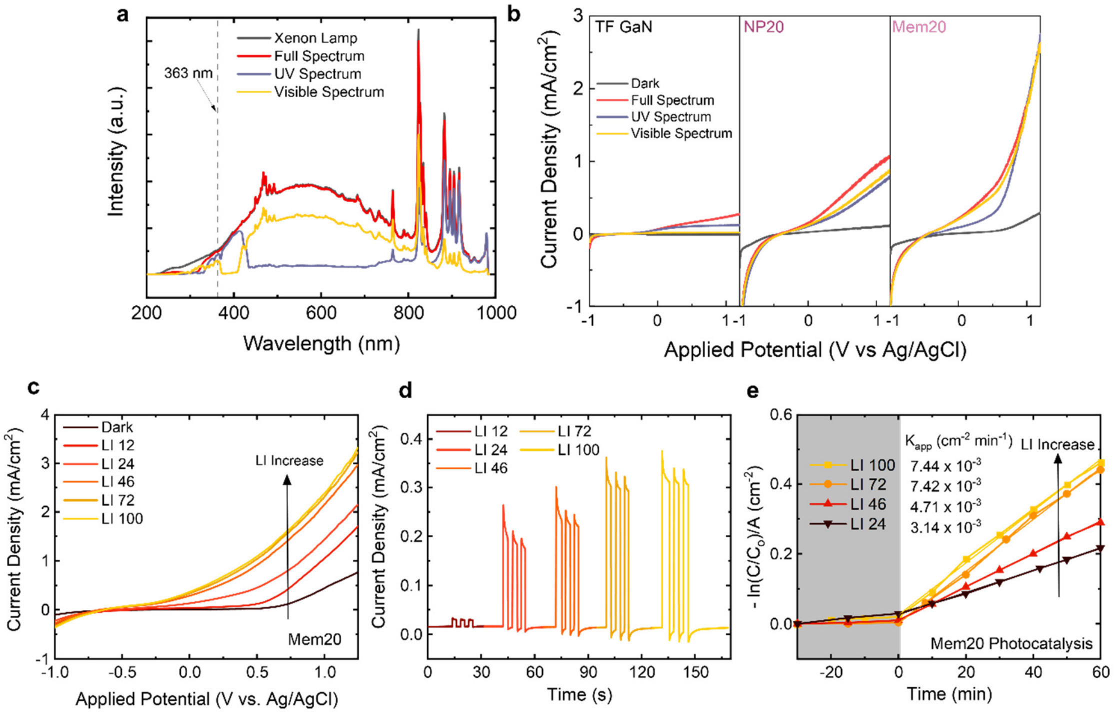

Figure S8. **(a) the illumination spectra of the original Xenon lamp and the spectra after the reflection of a mirror (full spectrum), a 405 nm short pass dichroic mirror (Visible spectrum), and a 405 nm long pass dichroic mirror (UV spectrum). (b) The LSV of the samples under different light illumination. (c) The LSV of the Mem20 under different full spectrum light intensities (LI) of 12, 24, 46, 72, and 100 mW/cm^2. (d) Photocurrent response of Mem20 under chopper illumination at different LI. (e) The apparent degradation rate constant fitting of Mem20 photocatalysis under different LI.**

Figure S8a, b shows the spectra of different light sources and the LSV of TF GaN, NP20, Mem20 sample under these light sources.

Figure S8c-e provide the laboratory test of Mem20 under different light intensity (LI) as supporting information for the rooftop measurement. The LI was tuned and calibrated to be 12, 24, 46, 72, and 100 mW/cm^2, where 24 – 72 mW is within the measured LI range during the rooftop field test. Under a weak illumination power (12 mW/cm^2), a tiny increase of the LSV and photocurrent response can still be observed, proving the excellent photon sensitivity of the Mem20. When enhancing the illumination power to 24-72 mW/cm^2, a clear LI-dependent photoresponse can be observed in **Figure S8c,d**. Indoor photocatalytic MB degradation by using Mem20 were conducted for 1 hour to mimic the natural environment for the rooftop tests. The corresponding apparent degradation rate constants (Kapp) were estimated, increasing from 3.14×10^{-3} to 7.42×10^{-3} cm^{-2}min^{-1}, as shown in **Figure S8e**. The photoresponse and MB

degradation rates are very close under the LI of 72 mW/cm^2 and 100 mW/cm^2. The main reason is the light absorption saturated because of the small thickness of the membrane (~ 1.2 μm in total).[5]

The actual Kapp in the following rooftop measurement can be higher than the indoor tests. On the one hand, it is because of the scaled-up wafer-size membrane. On the other hand, even though LI varied during the daytime, the quickly-bleached MB electrolyte (much lower MB concentration by the end of the day) allowed more photons to reach the membrane surface and contribute to accelerated photocatalytic MB degradation.

S9. Wafer-scale GaN membrane

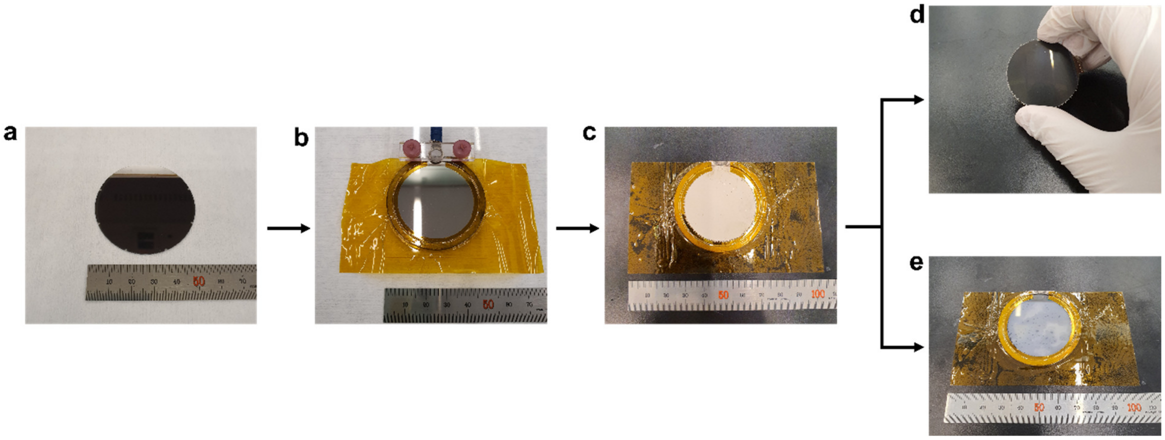

Figure S9. The fabrication process of the wafer-scale GaN membrane. (a) A planarized 2-inch n-GaN layer with NP20. (b) The 2-inch wafer with a seeding layer (50 nm-thick Ti and Ni). (c) Deposition of the Ni stressor. (d) a wafer-scale GaN membrane. (e) The substrate side after the wafer-scale exfoliation.

S10. Rooftop measurements

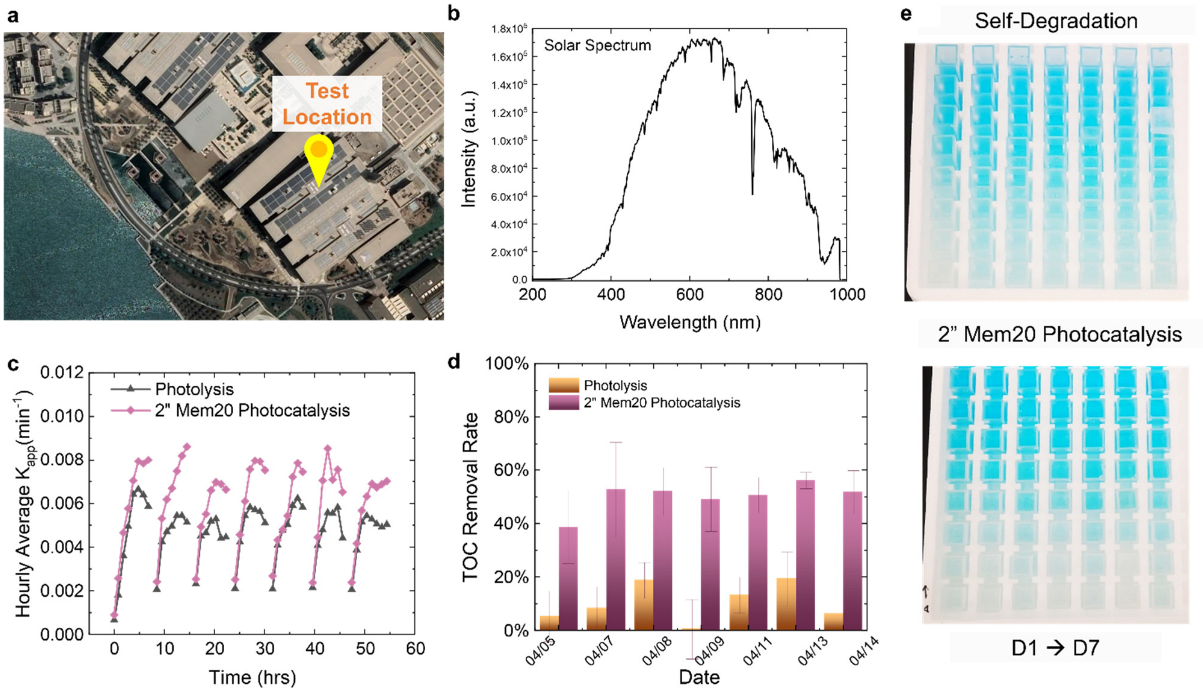

Figure S10. (a) Satellite view of the test location. (b) A representative solar spectrum is measured during the day. (c) hourly average k_{app}, (d) TOC removal rate, and (e) pictures of the sampling solution of photolysis and membrane photocatalysis.

The hourly average K_{app} were calculated by the following equation, assuming the reaction prone to the pseudo-first-order kinetic model in each hour.

$$Hourly\ Average\ K_{app} = \frac{d\ln\ln\frac{C_t}{C_0}}{dt} \qquad \text{Equation. S10}$$

References:

[1] a) M. Drygas, J. F. Janik, *Materials Chemistry and Physics* **2012**, *133* (2), 932, https://doi.org/https://doi.org/10.1016/j.matchemphys.2012.01.119; b) X. Luo, X. Zheng, D. Wang, Y. Zhang, H. Cheng, X. Wang, H. Zhuang, Y. Lou, *Sensors and Actuators B: Chemical* **2014**, *202*, 1010, https://doi.org/https://doi.org/10.1016/j.snb.2014.06.040.

[2] S. Strite, H. Morkoç, *Journal of Vacuum Science & Technology B: Microelectronics and Nanometer Structures Processing, Measurement, and Phenomena* **1992**, *10* (4), 1237, https://doi.org/10.1116/1.585897.

[3] a) B. Wang, T. Wang, A. Haque, M. Snure, E. Heller, N. Glavin, *Applied Physics Letters* **2017**, *111* (11), 113103, https://doi.org/10.1063/1.5002690; b) W.-C. Chen, S.-Y. Kuo, W.-L. Wang, J.-S. Tian, W.-T. Lin, F.-I. Lai, L. Chang, *Nanoscale Research Letters* **2012**, *7* (1), 468, https://doi.org/10.1186/1556-276X-7-468.

[4] E. Brillas, C. A. Martínez-Huitle, *Applied Catalysis B: Environmental* **2015**, *166-167*, 603, https://doi.org/https://doi.org/10.1016/j.apcatb.2014.11.016.

[5] E. Kusmierek, Semiconductor Electrode Materials Applied in Photoelectrocatalytic Wastewater Treatment—an Overview. In *Catalysts*, **2020**; Vol. 10.
